# FLAIR hyperintensity along the brainstem surface in leptomeningeal metastases: a case series and literature review

**DOI:** 10.1186/s40644-020-00361-8

**Published:** 2020-11-23

**Authors:** Koichi Mitsuya, Yoko Nakasu, Shoichi Deguchi, Kensei Shirata, Koiku Asakura, Kazuaki Nakashima, Masahiro Endo, Toshiaki Takahashi, Nakamasa Hayashi

**Affiliations:** 1 Division of Neurosurgery, Shizuoka Cancer Center, Nagaizumi, Shizuoka 4118777 Japan; 2 Division of Neurosurgery, Shiga University of Medical Science, Otsu, Shiga Japan; 3 Division of Diagnostic Radiology, Shizuoka Cancer Center, Nagaizumi, Shizuoka Japan; 4 Division of Thoracic Oncology, Shizuoka Cancer Center, Nagaizumi, Shizuoka Japan

**Keywords:** Brainstem, Diffusion MRI, FLAIR, Leptomeningeal metastasis, Non-small cell lung cancer, Targeted therapy

## Abstract

**Background:**

The incidence of leptomeningeal metastasis (LM) is underestimated because of its non-specific signs and the low sensitivity of clinical diagnostic modalities. Cerebrospinal magnetic resonance (MR) imaging with and without contrast enhancement (CE) is a gold standard for the neuroradiological assessment of patients with suspected LM. Previous studies suggested that some LM cases show changes of the brainstem surface on non-contrast MR images without or before the appearance of abnormalities on CE images. We assessed the features of this non-contrast MR finding in a cohort of LM patients in this retrospective single-institution study.

**Methods:**

We reviewed head MR images and clinical data of 142 consecutive patients in whom the final diagnosis was LM.

**Results:**

We found that 11 of these 142 patients (7.7%) with LM had band-like hyperintensity on the brainstem surface on non-enhanced FLAIR images, which looked like bloomy rind on cheese. Three of seven patients who were examined using diffusion-weighted imaging showed restricted diffusion in the corresponding lesion site. The above-mentioned 11 patients included 10 women and 1 man, with a median age of 61 years. All 11 patients had primary lung adenocarcinoma. Seven patients had symptomatic hydrocephalus. Ten patients had EGFR-mutated and one had ALK-rearrangement adenocarcinomas. Before the diagnosis of LM, 10 patients had undergone systemic therapy with EGFR-TKI or pemetrexed, and 1 patient with ALK inhibitor and bevacizumab.

**Conclusions:**

We present a series of patients with bloomy rind sign that is non-enhancing LM reliably detected by FLAIR hyperintensity on the brainstem surface. This finding is rare, but may reflect the spread of cancer cells in both the leptomeningeal membrane and the surface of the brain parenchyma specifically in patients with lung adenocarcinomas. Further study is needed to determine the clinical significance of this sign, and the pathophysiological factors associated with it may be clarified by analyzing serial MR images in a larger cohort of patients treated for LM.

## Background

Leptomeningeal metastasis (LM) is a complication of malignancy with dissemination of cancer cells to the pia mater, arachnoid membrane, and cerebrospinal fluid (CSF). Regarding the frequency of LM, various findings ranging from 5 to 10% of patients with solid cancers have been reported [1, 2]. The incidence of LM may increase with the longer survival of patients with cancer due to the recent development of effective systemic therapies. However, the prognosis is still poor after the diagnosis of LM. Early detection of leptomeningeal disease may allow an appropriate therapeutic plan to be implemented.

The gold standard for the diagnosis of LM is CSF cytology. In clinical practice, evaluation with gadolinium contrast-enhanced (GdCE) magnetic resonance (MR) imaging is most sensitive, and is often the first diagnostic choice for patients with suspected LM. However, CE-MR imaging yields up to 30% false-negative findings [2]. We therefore need more information from patients without abnormal GdCE-MR images.

A few case reports demonstrated abnormalities in fluid-attenuated inversion recovery (FLAIR) imaging of non-enhancing intracranial metastases including LM [3–10]. In particular, FLAIR hyperintensity in the brainstem surface, looking like a bloomy rind on cheese, was a peculiar finding shown to contribute to the detection of subtle LM in four previous studies [6, 7, 9, 10]. This abnormal finding may reflect both an early stage of cancer spread in the CSF space and also the progression of invasion in the brain surface.

In this study, we retrospectively reviewed MR images of our patients with LM to assess the incidence and the background features of FLAIR hyperintensity non-enhancing lesions on the brainstem surface, and speculate about their pathophysiological mechanism.

## Methods

Analysis of the medical records was performed after approval by the Institutional Research Ethics Board of Shizuoka Cancer Center (28-J173–28–1-3). Our ethics board waived the requirement for written informed consent for this retrospective observational study.

**Fig. 1:**
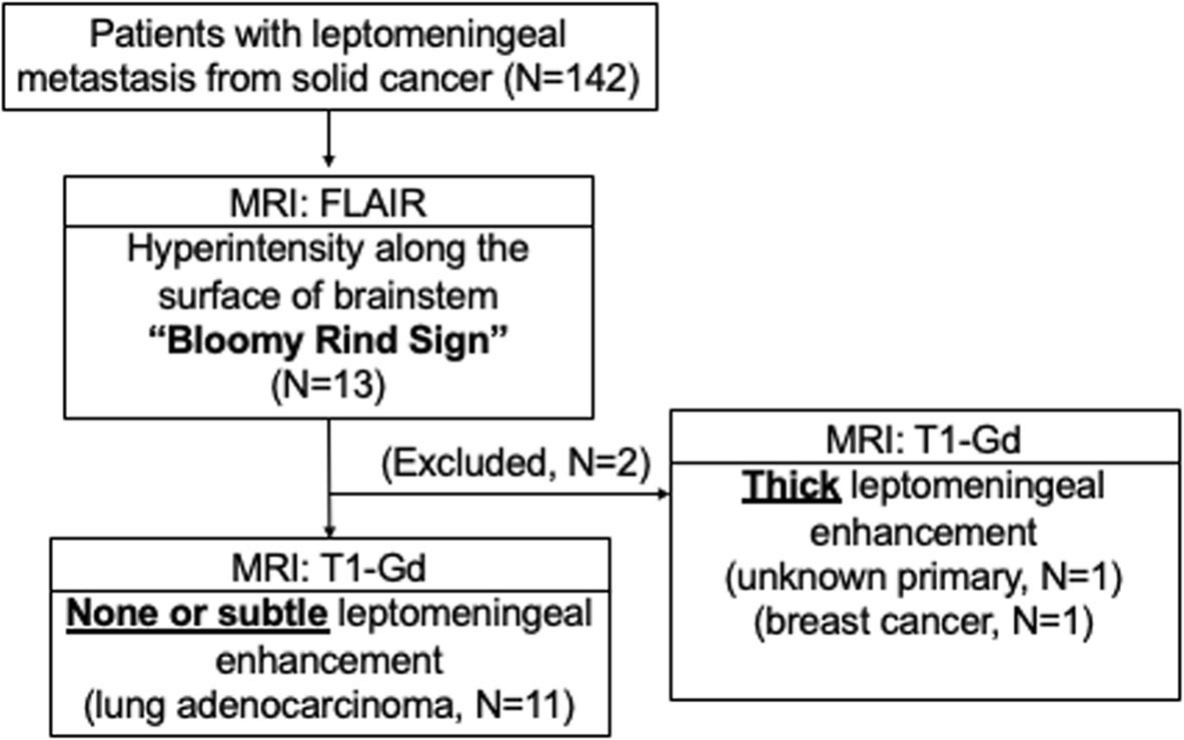
Flowchart showing the process of selection of the 11 cases in patients with leptomeningeal metastasis from solid cancer eligible for this study

After anonymizing the data, we reviewed the neurological records, survival data, corresponding plans of radiotherapy and systemic therapy, images, and independent pathological reports. We reviewed head MR images of 142 consecutive patients in whom the final diagnosis was LM during a period of 16 years (September 2002 to August 2018) (Fig. 1). Bloomy rind sign was picked up by two board-certified oncological neurosurgeons (> 10 years of experience in evaluation of neuro-oncological images). The image findings were visually confirmed by three board-certified radiologists (> 10 years of experience in neuro-imaging).

MR imaging was performed with either a 1.5Tesla or a 3Tesla system (Intera and Achieva dStream, Phillips, Best, Netherlands, or Genesis Signa, GE, IL).

The MR (1.5Tesla) imaging parameters were as follows: (1) FLAIR: TR/TE/TI 11000/100/2800 ms; NEX 2; matrix 304 × 219; thickness 5 mm; gap 0.5 mm; FOV 230 mm; (2) DWI: TR/TE 4000/70 ms; NEX 2; matrix 144 × 144; thickness 5 mm; gap 0.5 mm; b = 0 and 1000 s/mm^2^; FOV 230 mm; and (3) 3D-gradient echo T1WI: TR/TE, 7.2/3.5 ms; FA 15; NEX 1; matrix 256 × 256; thickness 4 mm; gap − 2 mm; FOV 230 mm.

The MR (3Tesla) imaging parameters were as follows: (1) FLAIR: TR/TE/TI 1100/125/2800 ms; NEX 1; matrix 400 × 235; thickness 5 mm; gap 0.5 mm; FOV 230 mm; (2) DWI: TR/TE 5000/60 ms; NEX 2; matrix 160 × 160; thickness 5 mm; gap 0.5 mm; b = 0 and 1000 s/mm^2^; FOV 230 mm; and (3) 3D-gradient echo T1WI: TR/TE 4.0/1.8 ms; FA 12; NEX 1; matrix 240 × 396; thickness 4 mm; gap − 2 mm; FOV 230 mm.

As contrast medium, meglumine gadopentetate (0.2 ml/kg) was used until October 2015 and gadobutrol (0.1 ml/kg) since November 2015.

Final diagnosis of LM was given by neurological examination, CSF cytology or radiographic evaluation according to RANO response criteria for LM [11]. CSF cytology was positive in 104 cases.

## Results

We found that 13 of 142 patients (9.2%) with LM had band-like FLAIR hyperintensity on the brainstem surface. Eleven (7.7% of the total) of these 13 patients had none or only faint abnormalities of the lesions on GdCE MR images, whereas the two other patients were excluded because of definitely abnormal enhancement of the leptomeninges: one patient with cancer of unknown origin and the other with breast cancer (Fig. 1). The age of the 11 patients ranged from 46 to 72 years (median 61 years), and there were 10 women and 1 man. Definitive diagnosis was made by cytological examination of CSF in all patients except one. Nine patients (9/11 = 81.8%) had symptomatic hydrocephalus that needed CSF shunt surgery (Table 1).

**Table 1:** Patient characteristics

	No (%)
Patients	11
Median age (range) years	60 (46–72)
Gender	
Male	1 (9)
Female	10 (91)
Primary cancer	
Lung adenocarcinoma	11 (100)
Driver mutation	
EGFR mutant	10 (91)
*19 del*	*5*
*21L858R*	*4*
*NA (NoSmokFem, supporsed to be positive)*	*1*
ALK rearrangement	1 (9)
Area of FLAIR hyperintensity (Bloomy Rind Sign)	
Pons	9 (82)
Midbrain and pons	1 (9)
Pons and medulla	1 (9)
GdCE T1W images at the same lesion	
No abnormality	6 (55)
Subtle abnormality	5 (45)
DW images at the same lesion	
Restricted diffusion	3 (27.2)
No abnormality	4 (36.4)
NA	4 (36.4)
Treatment regimen before LM	
Gefitinib	3 (27.25)
Gefitinib, Pemetrexed+/−Pratinum	3 (27.25)
Erlotinib, Gefitinib, Pemetrexed	1 (9)
Erlotinib, Pratinum+Pemetrexed, Taxane	1 (9)
Osimertinib	1 (9)
Pratinum+Taxane+Bevacizumab, Pemetrexed	1 (9)
Alectinib, Platinum+Taxane+Bevacizumab	1 (9)
Symptomatic hydrocephalus	
Yes	7 (64)
No	4 (36)
Brain parenchymal metastasis	
Yes	9 (82)
No	2 (18)

*NA* Not available

All 11 patients had primary lung adenocarcinoma. Ten patients had epidermal growth factor receptor (EGFR)-mutated and one had anaplastic lymphoma kinase (ALK)-rearrangement adenocarcinomas. Before the diagnosis of LM, ten patients had undergone systemic therapy with EGFR-tyrosine kinase inhibitor (TKI) or pemetrexed, and the patient with ALK rearrangement had undergone treatment with systemic ALK inhibitor and bevacizumab (Table 1).

**Fig. 2:**
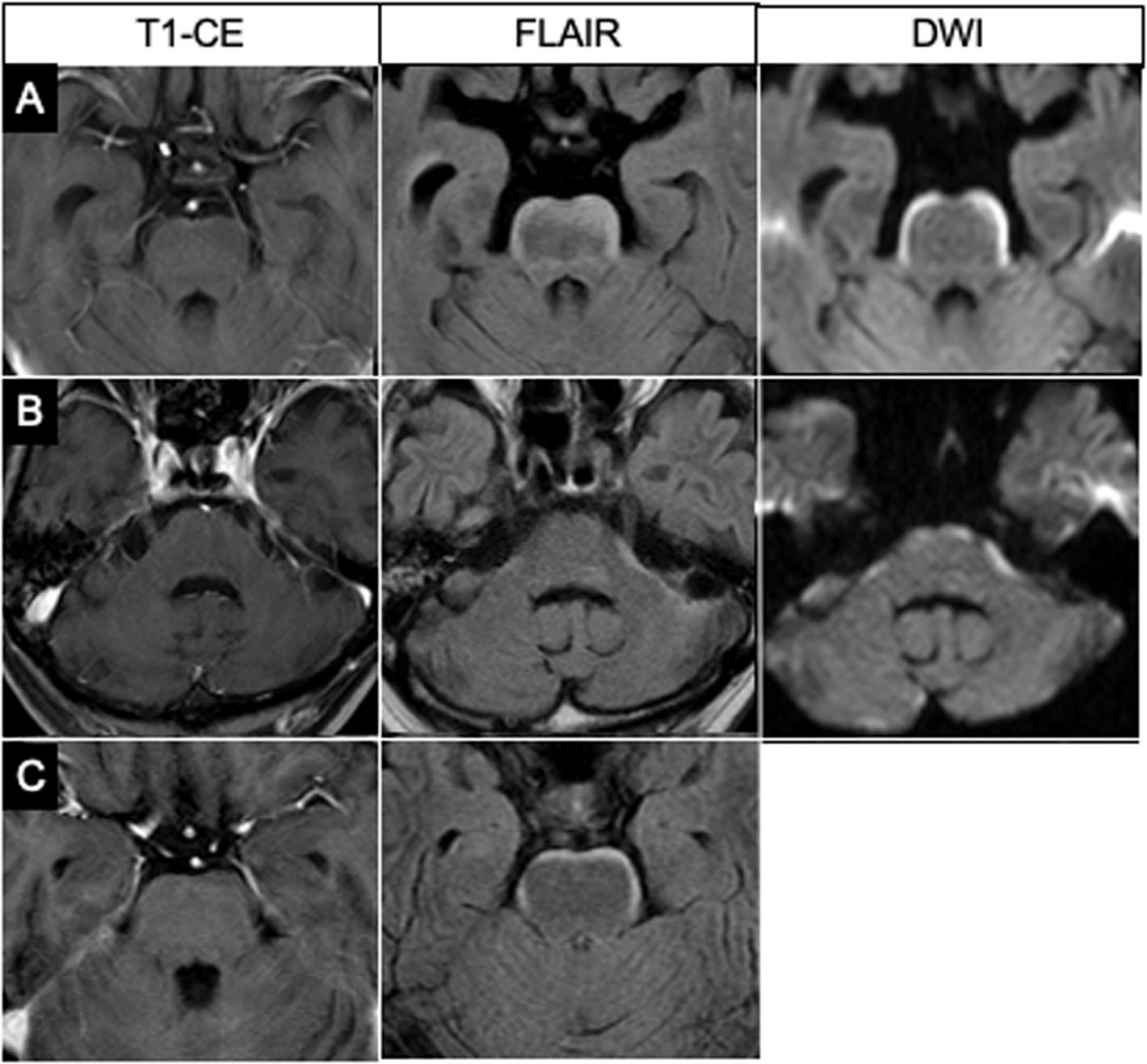
Three representative cases of bloomy rind sign. Although no abnormalities are seen on the brainstem on GdCE T1-weighted images, FLAIR images show hyperintensity along the brainstem surface in all three cases. Figure 2**a** and **c** show FLAIR hyperintensity on the antero-lateral surface of the pons, and Fig. 2**b** shows it along the antero-lateral cerebellar peduncle. DW images show restricted diffusion at the same lesions of two patients. DW images are not available for the third patient. Two patients (Figs. **a** and **c**) had positive CSF cytology, but one (Fig. **b**) did not undergo CSF examination because of neurological signs and restricted enhancement in the internal acoustic canals on GdCE images

FLAIR images showed a hyperintense band along the anterior and lateral surfaces of the brainstem extending to the cerebellar peduncle, even to the walls of the fourth ventricle. It was a clear and dense layer on the surface of the brainstem looking like a bloomy rind on cheese, symmetrical in all 11 cases, and had some accentuation or interruption (Fig. 2). Overall, 6 of the 11 cases (54.5%) showed no abnormal enhancement of the brainstem, but five had only subtle enhancement on part of the lesion on GdCE MR images. Diffusion-weighted (DW) imaging was performed for 7 of the 11 patients and demonstrated the surface lesions as having restricted diffusion in 3 patients and normal in 4 of them (Table 1).

In this study, 10 of 11 patients had positive CSF cytology, and remaining one did not undergo CSF cytological examination because of definitive neurological sings and MR findings. From another point of view, CSF cytology was positive in 104 patients:10 patients showed both positive CSF cytology and bloomy rind sign, however, 94 patients showed positive CSF cytology but no bloomy rind sign.

All 11 patients had received systemic therapy for lung cancer before the diagnosis of LM with bloomy rind sign: 9 with an EGFR-TKI, one with an EGFR-TKI plus an anti-angiogenic antibody, and another with cytotoxic anti-cancer drugs plus an anti-angiogenic antibody (Table 1).

### Representative case

**Fig. 3:**
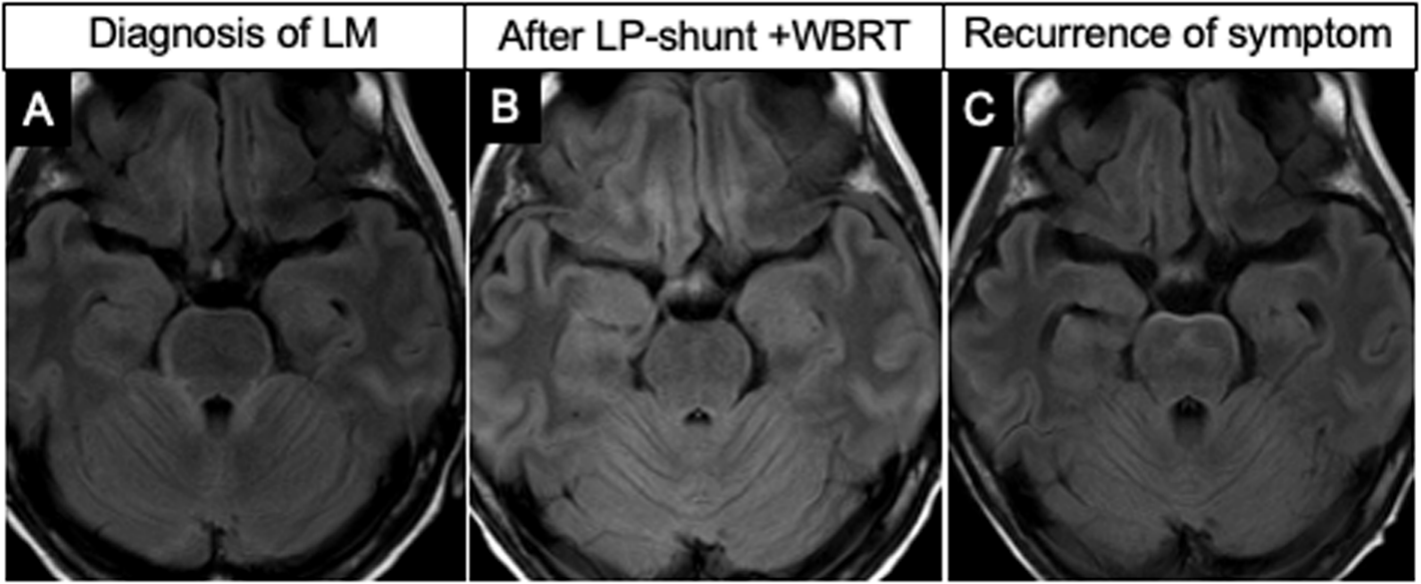
FLAIR images show the bloomy rind sign (Fig. 3**a**) and demonstrate its disappearance after LP-shunting and WBRT (Fig. 3**b**). A subsequent brain MR image shows its reappearance with deteriorated neurological symptoms 2 months later (Fig. 3**c**)

A woman was diagnosed with lung adenocarcinoma and underwent chemotherapy with gefitinib. She was admitted to our hospital with headache and nausea. Brain MR images showed a hyperintense lesion along the brainstem surface on FLAIR and DW images (Fig. 3a). There were no enhancing lesions with GdCE in the brain parenchyma or leptomeninges. Cytological examination of lumbar spinal fluid revealed malignant cells. She underwent lumbar-peritoneal shunt surgery followed by whole-brain radiation therapy, which improved her symptoms. Brain MR images demonstrated disappearance of the hyperintense lesion on FLAIR at this stage (Fig. 3b). However, 2 months later, she was admitted with deteriorated neurological symptoms. Brain MR images showed a non-enhancing, FLAIR hyperintensity lesion along the brainstem surface again (Fig. 3c).

## Discussion

LM may increase in incidence with the longer survival of patients with solid cancer due to the development of effective systemic therapies. However, the prognosis is still poor after the diagnosis of LM, and individualized treatment have not been established. Early detection of LM by non-invasive measures would enhance optimal clinical approaches to this devastating disease. In this retrospective study, we demonstrated that FLAIR imaging as a potentially simple method for early detection of LM specifically in patients with non-small cell lung cancer (NSCLC).

FLAIR hyperintensity on the brainstem surface without abnormal enhancement is a characteristic but rare finding as we retrospectively identified it in only 11 (7.7%) of 142 patients with known LM at our institute. We called this characteristic layer of hyperintensity a bloomy rind sign. All patients had LM from lung adenocarcinoma, they had undergone chemotherapy mainly with TKIs, and five patients had also undergone whole-brain radiotherapy before presenting LM.

Reviewing the literature, we identified nine cases with similar MR findings, all of which had lung adenocarcinoma [6, 7, 9, 10]. The clinical characteristics of the nine cases are summarized in Table 2. There were five males, one female, and three others of unknown sex, with ages ranging from 39 to 77 years. All of the cases had NSCLC as well as our 11 cases. Seven cases had bone, lung, or brain parenchymal metastasis. Six cases had confirmed EGFR gene mutations and the other three cases had not undergone mutational analysis. In one patient, the FLAIR hyperintensity of the brainstem led to the diagnosis of primary lung cancer [9]. Five cases had taken an EGFR-TKI before diagnosis of the LM. Five cases had malignant cells in CSF and three others were associated with brain parenchymal metastasis. The brain lesions were treated with radiotherapy in three cases, intrathecal chemotherapy in one, and EGFR-TKIs in three.

**Table 2:** Previous reports of FLAIR hyperintensity on the brainstem surface without abnormal enhancement (Bloomy rind sign)

Case	Author, publish year	Age	Sex	Primary cancer	EGFR mutation	Systemic metastasis	Chemotherapy agents used before LM	CSF cytology	Treatment for LM	Prognosis
1	Crombe, 2014 [6]	56	M	Lung adenocarcinoma	Positive	Bone, Lung	gefitinib	Malignant cells	NA	2 M (Respilatory fail)
2	Khil et al. 2015 [7]	75	M	Lung adenocarcinoma	NA, but supposed to be positive^a^1	Bone	gefitinib	Malignant cells	NA	1 M (Respilatory fail)
3	Khil et al. 2015 [7]	47	F	Lung adenocarcinoma	NA	Brain	CBDCA+PTX	NA	WBRT	Hospice care
4	Cheng, 2017 [9]	39	M	Lung adenocarcinoma	Positive (19 del)	Lung	CDDP+VNR, gefitiib	Malignant cells	IT-Ig, steroid, IT-CTx, and erlotinib	1Y (Sepsis)
5	Cheng, 2017 [9]	NA	NA	Lung adenocarcinoma	Positive	Brain, Lung	CDDP+VNR, gefitiib	NA	WBRT	Lost to follow up
6	Cheng, 2017 [9]	NA	NA	Lung adeno+squamous	Positive (21 L858R)	Bone, Lung	none	negative	gefitinib, afatinib	7 M (Respilatory fail)
7	Cheng, 2017 [9]	NA	NA	Lung adenocarcinoma	Positive (19 del, T790M)	Bone, Brain, Lung	erlotinib, VNR, pemetrexed, and gemcitabine	NA	WBRT, osimertinib	survival over 6 M
8	Maeda, 2019 [10]	55	M	Lung adenocarcinoma	Positive (19 del)	NA	NA	Malignant cells	NA	spread to medulla after 4 M
9	Maeda, 2019 [10]	77	M	Lung adenocarcinoma	NA	NA	NA	Malignant cells	NA	NA

^a^1, The author did not mention if there was EGFR mutation, but the patient received gefitinib

*NA* Not available; *CBDCA* Carboplatin; *CDDP* Cisplatin; *VNR* Vinorelbine; *WBRT* Whole brain radiotherapy; *IT* Intrathercal; *CTx* Chemotherapy; *Ig* Immunoglobulin

*PTX* Paclitaxel

For detecting LM, FLAIR images reportedly had a specificity as high as GdCE T1-weighted images, although they had low sensitivity [12, 13]. For the detection of non-enhancing LM, FLAIR hyperintensity of the brainstem surface may contribute to the diagnosis of patients as a non-invasive diagnostic measure. As for a definitive diagnosis, non-enhancing images may need to be improved in terms of their sensitivity and used in combination with other diagnostic tools.

Although the mechanism behind the FLAIR hyperintensity of the brainstem surface without abnormal enhancement in LM remains unclear, the results of our study provide information for understanding the pathophysiology of this disease. First, T1-weighted MR imaging may have a limitation in terms of its spatial resolution for detecting subtle enhancement of thin layers along the brainstem surface [8]. Disturbances in the blood-brain or blood-CSF barrier and vascularization may vary in degree and distribution on the thin surface around the brainstem. Second, our study showed restricted diffusion in three of seven patients examined by DW imaging. That may reflect cytotoxic edema involving the brainstem surface, and may also reflect microinfarction due to vascular injury by invaded tumor cells [5, 7]. Third, the restricted diffusion may also be caused by highly cellular infiltrating cancer in the subarachnoid and subpial spaces of the brainstem [9]. Fourth, targeted therapy may result in the regression of microvessels and inhibition of neovascularization, leading to less of an enhancement effect of active cancer on MR images [4, 8, 14]. Fifth, the findings in seven patients harboring hydrocephalus in our study suggested retention of CSF in isolated subarachnoid spaces and infiltration into the brainstem surface. One patient had severe hydrocephalus and her abnormal FLAIR hyperintensity was reversible in accordance with her good clinical response to lumbar-peritoneal CSF shunt surgery (Fig. 3).

Increasing use of anti-angiogenic and immune-modulating drugs for systemic cancers may modify the diagnostic sensitivity of MR images with GdCE T1-weighted imaging. Closer examination with FLAIR and DW images may support early detection of the devastating disease of LM.

### Limitations

Although unenhanced FLAIR images reportedly have sufficient specificity for detecting LM [13], we have to carefully interpret the leptomeningeal hyperintensity and differentiate LM from other pathological conditions such as subarachnoid hemorrhage, bacterial meningitis, and low intracranial pressure. Other limitations in this retrospective study include that the number of cases was limited and that MR imaging was performed using different instruments and sequences. In addition, DW imaging was performed only in selected patients. We need more information of this rare and subtle abnormality with better resolution. Finally, patients with LM were not sufficiently followed up with frequent MR imaging in their courses of difficult treatment.

## Conclusions

In this paper, we have presented a series of patients with non-enhancing LM that was detected by FLAIR hyperintensity on the brainstem surface. This bloomy rind sign is rare, but may reflect both the early stage of LM and progressive infiltration into the brain surface. Non-contrast sequences should be carefully examined when evaluating LM in patients with NSCLC, especially those with a history of TKI therapy. The mechanism behind this sign remains unclear, but we hypothesized that it involves various pathological processes. Further study is needed to determine the clinical significance of this sign and the factors correlated with it. Prospective analysis of serial MR images in patients with LM from NSCLC may provide clues to understand the mechanism and to improve the available therapeutic options.,

## Data Availability

No additional data.

## Abbreviations

ALK: Anaplastic lymphoma kinase
CSF: Cerebrospinal fluid
DW: Diffusion-weighted
EGFR: Epidermal growth factor receptor
FLAIR: Fluid -attenuated inversion recovery
GdCE: Gadolinium contrast enhancement
LM: Leptomeningeal metastasis
MR: Magnetic resonance
NSCLC: Non-small cell lung cancer
TKI: Tyrosine kinase inhibitor
